# The volatilome reveals microcystin concentration, microbial composition, and oxidative stress in a critical Oregon freshwater lake

**DOI:** 10.1128/msystems.00379-23

**Published:** 2023-08-17

**Authors:** Lindsay Collart, Duo Jiang, Kimberly H. Halsey

**Affiliations:** 1 Department of Microbiology, Oregon State University, Corvallis, Oregon, USA; 2 Department of Statistics, Oregon State University, Corvallis, Oregon, USA; University of Illinois at Chicago, Chicago, Illinois, USA

**Keywords:** cyanobacteria, volatile organic compounds, microcystin, harmful algal blooms, cyanotoxins, saturated fatty aldehydes, HAB monitoring

## Abstract

Toxins produced by cyanobacterial blooms in freshwater lakes are a serious public health problem. The conditions leading to toxin production are unpredictable, thereby requiring expensive sampling and monitoring programs globally. We explored the potential of volatile organic compounds (VOCs) to indicate microcystin presence and concentration and microbial community composition in Upper Klamath Lake, Oregon. Elastic net regularization regression selected 24 of 229 detected *m/z +* 1 values (corresponding to unique VOCs) in models predicting microcystin contamination. These models outperformed regression models based only on environmental parameters, including chlorophyll, pH, and temperature. Several *m/z +* 1 values selected by elastic net were putatively identified as saturated fatty aldehydes, which defend cyanobacteria against oxidative stress. Elastic net also identified unique sets of *m/z +* 1 values that predicted the relative abundance of the dominant bacterial phyla, classes, and cyanobacterial genera. VOCs appear to reveal the physiological status of cyanobacteria during toxic blooms and may be a key component of lake monitoring strategies.

**IMPORTANCE:**

Harmful algal blooms are among the most significant threats to drinking water safety. Blooms dominated by cyanobacteria can produce potentially harmful toxins and, despite intensive research, toxin production remains unpredictable. We measured gaseous molecules in Upper Klamath Lake, Oregon, over 2 years and used them to predict the presence and concentration of the cyanotoxin, microcystin, and microbial community composition. Subsets of gaseous compounds were identified that are associated with microcystin production during oxidative stress, pointing to ecosystem-level interactions leading to microcystin contamination. Our approach shows potential for gaseous molecules to be harnessed in monitoring critical waterways.

## INTRODUCTION

Cyanobacterial harmful algal blooms (cyanoHABs) occur globally and are characterized by excessive growth of photosynthetic bacteria in freshwater lakes and rivers. CyanoHABs degrade water quality, negatively impacting potability, aquatic life, and agricultural and recreational activities. Some cyanoHABs produce toxins that pose direct threats to animal and human health (1 – 5) and also stunt crop development (6, 7). The chemical ecology of cyanotoxins is not well understood, but they appear to provide grazing defense and protection from reactive oxygen species (ROS) that can stimulate cyanobacterial growth and production (8). Cyanotoxins can also alter the microbial community and disrupt multitrophic interactions (9). Annual economic losses caused by cyanoHABs in the USA alone are conservatively valued at $2–$4 billion (10, 11), and the severity and consequences of cyanoHABs are predicted to be exacerbated by climate change (12 – 16). These widely ranging impacts call for near to real-time monitoring of cyanobacteria and their toxins to protect the public and effectively manage cyanoHABs in source and recreational waters (17).

CyanoHAB monitoring programs are challenged because the specific toxins produced are strain-specific, and no morphological shifts or commonly measured environmental triggers are known to be reliably associated with toxin production (17). Quantifying the genes encoding cyanotoxins in an ecosystem offers one approach to assess the risk of cyanotoxin contamination, but may be of limited value because the presence of cyanotoxin genes is not evidence of its expression (18). Direct cyanotoxin measurement in water and fish tissues by enzyme-linked immunosorbent assay (ELISA) and liquid chromatography-tandem quadrupole mass spectrometry (LC-MS/MS) (19) is expensive, specific to a subset of congeners (20) and cannot identify the producer or its abundance (21). Tools to leverage high-resolution detection of cyanobacterial cells and their metabolites remain nascent in application but are needed to address many environmental problems that are reaching ecological tipping points despite decades of intensive scientific effort (22).

We investigated the potential of the volatilome to provide high-sensitivity detection of cyanoHABs and cyanotoxin production (23 – 25). The “volatilome” is the full range of low-molecular-weight (~30–272 a.m.u.) volatile organic compounds (VOCs) produced in an ecosystem (26). VOCs have roles in cell signaling (27 – 30), predator-prey interactions (31), microbial carbon cycling (32, 33), and atmospheric emissions that impact tropospheric ozone and climate (34). Some VOCs inhibit growth and induce lysis in algal community members (35 – 37), thus regulating microbial interactions and community composition (37 – 39). VOC production depends on the algal species present, their growth phase, and their environment (29, 32, 40 – 42).

Algae, including cyanobacteria, release a wide array of VOCs, including terpenes, fatty acids and their 2-keto acid degradation products, alkanes and alcohols (38, 43 – 45) as a result of primary and secondary metabolism (46, 47) and indirectly through photochemical reactions with dissolved organic matter (48). Cyanobacteria produce medium-chain aliphatic hydrocarbons that strengthen and add flexibility and fluidity to lipid bilayers (49, 50), as well as provide tolerance to temperature and light stress (51, 52). Alkanes (C_*n*_) and their corresponding alcohols and aldehydes are produced from C_*n* + 1_ fatty aldehydes via aldehyde deformylating oxygenase (ADO) yielding aliphatic hydrocarbons of varying lengths (51). The array of volatile hydrocarbons present in cyanobacteria appears to provide mechanisms for managing cell-level oxidative stress (51).

Upper Klamath Lake (UKL) is a large shallow lake in southern Oregon that is a hub of complex water use for agriculture, wildlife, fisheries, recreation, and Tribal subsistence and culture. Intensive farming and drought have decreased water quality in UKL over the last half century, contributing to annual cyanoHAB events. UKL cyanoHABs are typically dominated by *Aphanizomenon* and *Microcystis* and produce the hepatotoxin, microcystin, at elevated concentrations that prompt public warnings to avoid water contact in the mid- to late-summer (53). We characterized the volatilome using proton transfer reaction time-of-flight mass spectrometry (PTR-ToF-MS) at lake and canal sites in UKL over 2 years and identified over 200 *m/z +* 1 values, corresponding to unique VOCs. Elastic net regularized regression selected small subsets of the *m/z +* 1 values that were effective predictors of microcystin contamination or microbial community composition in UKL. Microcystin prediction by elastic net models outperformed other models based only on environmental variables and in-water properties that are commonly used to detect cyanoHAB development. Several *m/z +* 1 values recurring in our elastic net models appear to be associated with the fatty aldehyde ADO pathway, suggesting these cyanobacterial metabolites underlie lipid repair and ROS reduction during oxidative stress, which is thought to be associated with microcystin production. These fatty aldehydes in combination with other key VOCs may indicate ecosystem interactions associated with microcystin production and represent important targets for cyanoHAB monitoring.

## RESULTS AND DISCUSSION

### Upper Klamath Lake chemical and microbial composition

**Fig 1:**
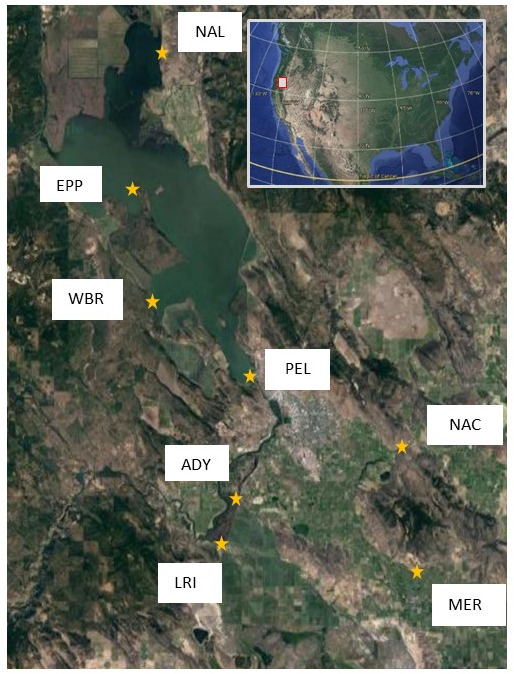
Sample sites on Upper Klamath and Agency Lakes, Oregon. Lake sites and their geospatial positions were NAL (42.559839–121.929579), WBR (42.314529–121.942224), EPP (42.430715–121.962764), and PEL (42.2390–121.8097). Canal sites and their geospatial positions were NCA (42.1222–121.8289), ADY (42.0808–121.8456), MER (42.0536–121.6006), and LRI (42.1733–121.6175).

**Fig 2:**
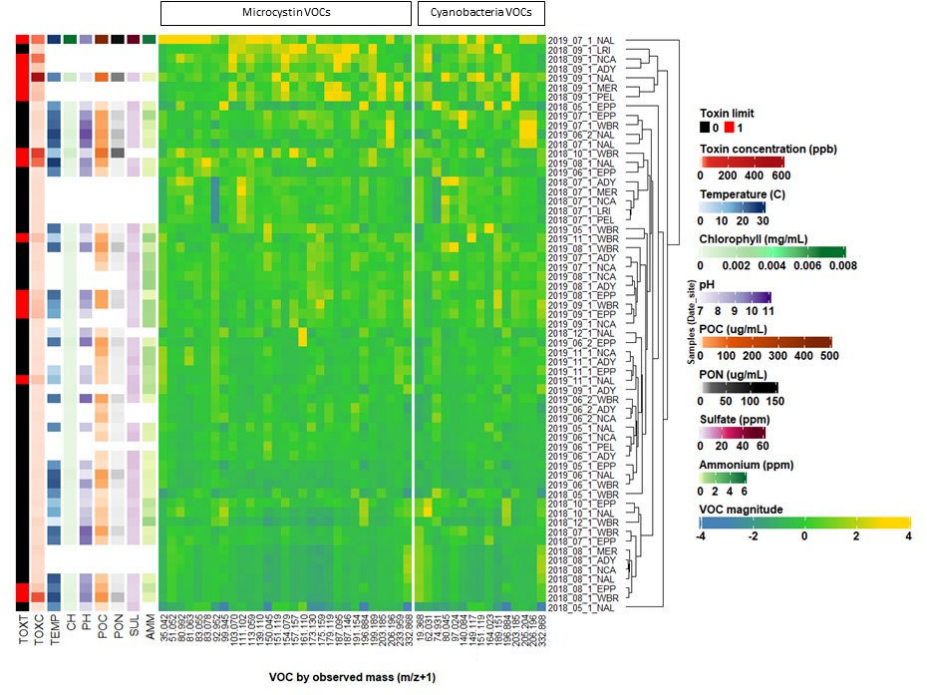
Unsupervised hierarchical clustering of *m/z +* 1 values selected by elastic net in M1, M2, M7, and M8 (left panel) and in models predicting cyanobacterial genera relative abundances (right panel) in lake and canal samples. Samples are shown in rows and labeled with site and date. The heatmap shows the Pearson correlation between each *m/z +* 1 value and microcystin concentration or relative abundances of cyanobacteria genera, with yellow being the most positively correlated and dark blue being the most negatively correlated (legend: VOC magnitude). To the left of the heatmap are environmental parameters identified by elastic net or stepwise linear regression or logistical models associated with each sample: TOXT, microcystin concentration ≥0.3 ppb (red) or <0.3 ppb (black); TOXC, microcystin toxin concentration (ppb); TEMP, temperature (°C); CH, chlorophyll concentration (mg/mL); pH; POC, particulate organic carbon (μg/mL); PON, particulate organic nitrogen (μg/mL); AMM, ammonium (ppm); and SUL, sulfate (ppm).

Water samples were collected from three sites on UKL, one site on its northern arm, Agency Lake, and four canal sites during the months of May–December in 2018 and 2019 (Fig. 1). The mean microcystin concentration among UKL samples with detectable toxin was 8.7 ppb (Table 1), surpassing the U.S. Environmental Protection Agency’s recommended health advisory limit for drinking water of 0.3 ppb for pre-school-aged children and 1.6 ppb for children and adults (54). The minimum reporting, recreational, and drinking water limits for microcystin vary by state depending on water use and potential for exposure (55). Of the 70 samples collected over 2018–2019, 10 UKL samples and three canal samples were contaminated with microcystin at concentrations ≥0.3 ppb. Toxic samples mostly occurred in summer months (July to Sept), but occasionally in November 2019, and occurred at all four lake sites (NAL, WBR, EPP, PEL) (Fig. 2). The highest microcystin concentration was 469 ppb from NAL in September 2019. Environmental parameters varied widely in UKL (Fig. 2; Table 1), and toxic samples were sometimes associated with high temperature, chloride, pH, particulate organic carbon (POC), particulate organic nitrogen (PON), chlorophyll, ammonium (AMM) and conductivity; however, no significant correlations were observed with microcystin concentration and any single parameter measured at UKL (Fig. S1).

**TABLE 1:** Environmental parameters collected at Upper Klamath Lake, Oregon^*a*^

Environmental parameter	Abbreviation		Minimum	Maximum	Mean	SD
Microcystin (ppb)	TOX		0	469.51	8.72	56.03	
*Chlorophyll (µg/mL*)	CH		0	6.8	0.24	0.94	
*Temperature (°C*)	TEMP		−0.1	26.87	17.62	6.65	
*pH*	PH		7.22	10.22	8.59	0.94	
*Conductivity (S/m*)	COND		61.4	133.6	102.9	12.6	
Particulate organic carbon (µg/mL)	POC		0.45	432.32	18.38	65.53	
Particulate organic nitrogen (µg/mL)	PON		0.066	100.20	3.96	15.12	
Chloride (ppm)	CHL		2.392	50	4.39	6.94	
Sulfate (ppm)	None		1.70	50	4.46	6.97	
Nitrate (ppm)	None		0.2	15.54	0.73	2.26	
Phosphate (ppm)	None		0.11	50	1.37	7.35	
Ammonium (ppm)	AMM		0.01	5.43	0.41	0.82	

^*a*^ Microcystin concentration is the first listed environmental parameter. Below microcystin, “low-cost” parameters are italicized and “high-cost” parameters are in normal font.

**Fig 3:**
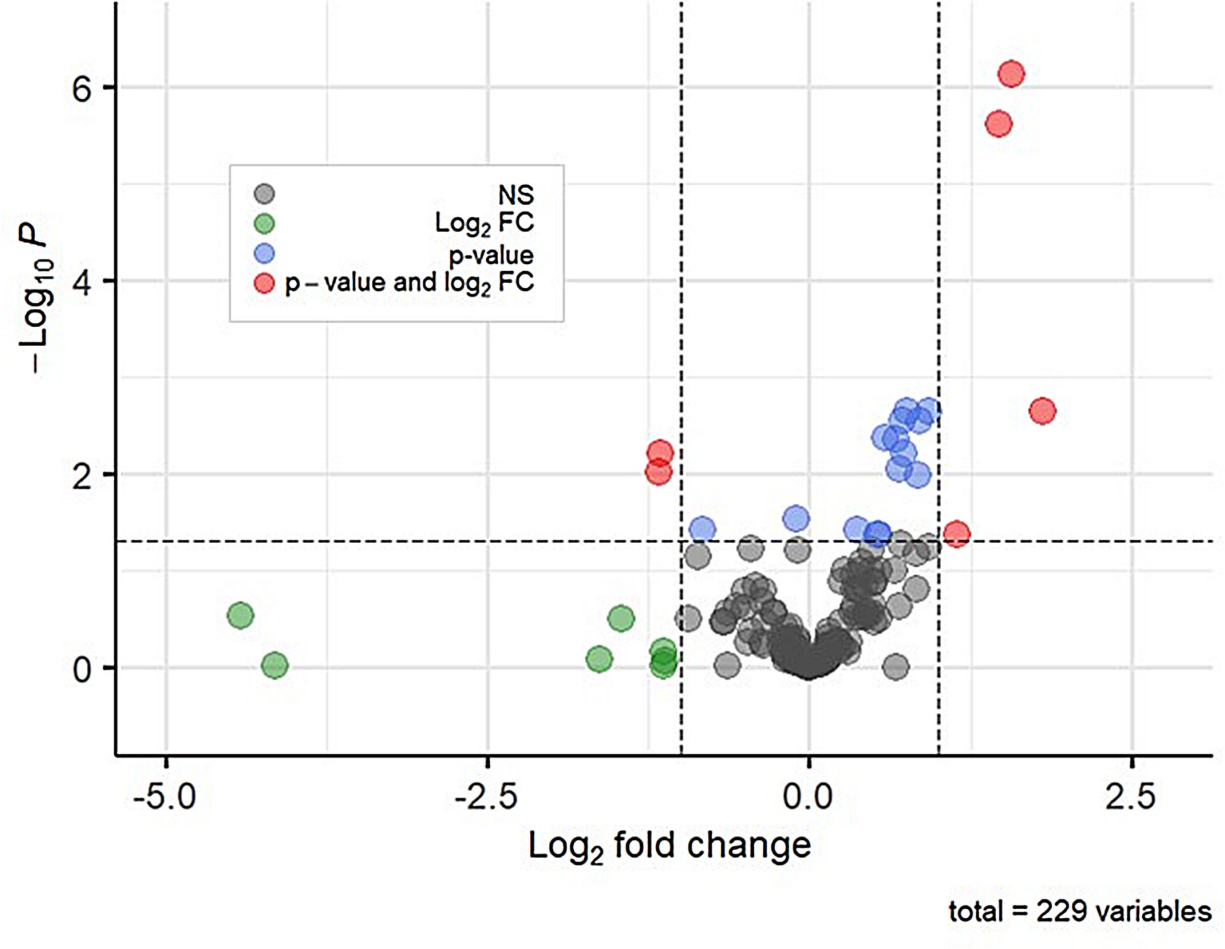
Volcano plot showing log_2_ fold changes (FC) and *P*-values (significance determined via Wald test) for the 229 *m/z +* 1 values in toxic (≥0.3 ppb) versus non-toxic samples. Multiple test correction using the Benjamin-Hochberg false discovery rate was applied to the *P*-value for each *m/z +* 1 value. The points are colored according to log_2_ fold changes and degree of significance. Points with positive fold changes greater than the dashed vertical line at +1.0 are *m/z +* 1 values enriched in toxic versus non-toxic samples. Points with negative fold changes less than the dashed vertical line at −1.0 are *m/z +* 1 values depleted in toxic versus non-toxic samples. NS, *m/z* + 1 value that is not present in significantly different amounts between toxic and non-toxic samples.

Untargeted volatilomics detected 229 *m/z +* 1 values in samples collected at UKL and associated canals during 2018 and 2019. Six *m/z +* 1 values were present in significantly discriminating amounts between samples with microcystin ≤0.3 ppb and samples with microcystin ≥0.3 ppb (Fig. 3). Using these six *m/z +* 1 values in a multiple linear regression model failed to predict microcystin contamination or concentration (*R* ^2^ = 0.08; *P*-value = 0.89). Volatilomes clustered well by sampling date, and samples collected in 2018 mostly clustered separately from those collected in 2019 (Fig. S2). Volatilomes of toxic samples did not demonstrate clear clustering (Fig. S2).

**Fig 4:**
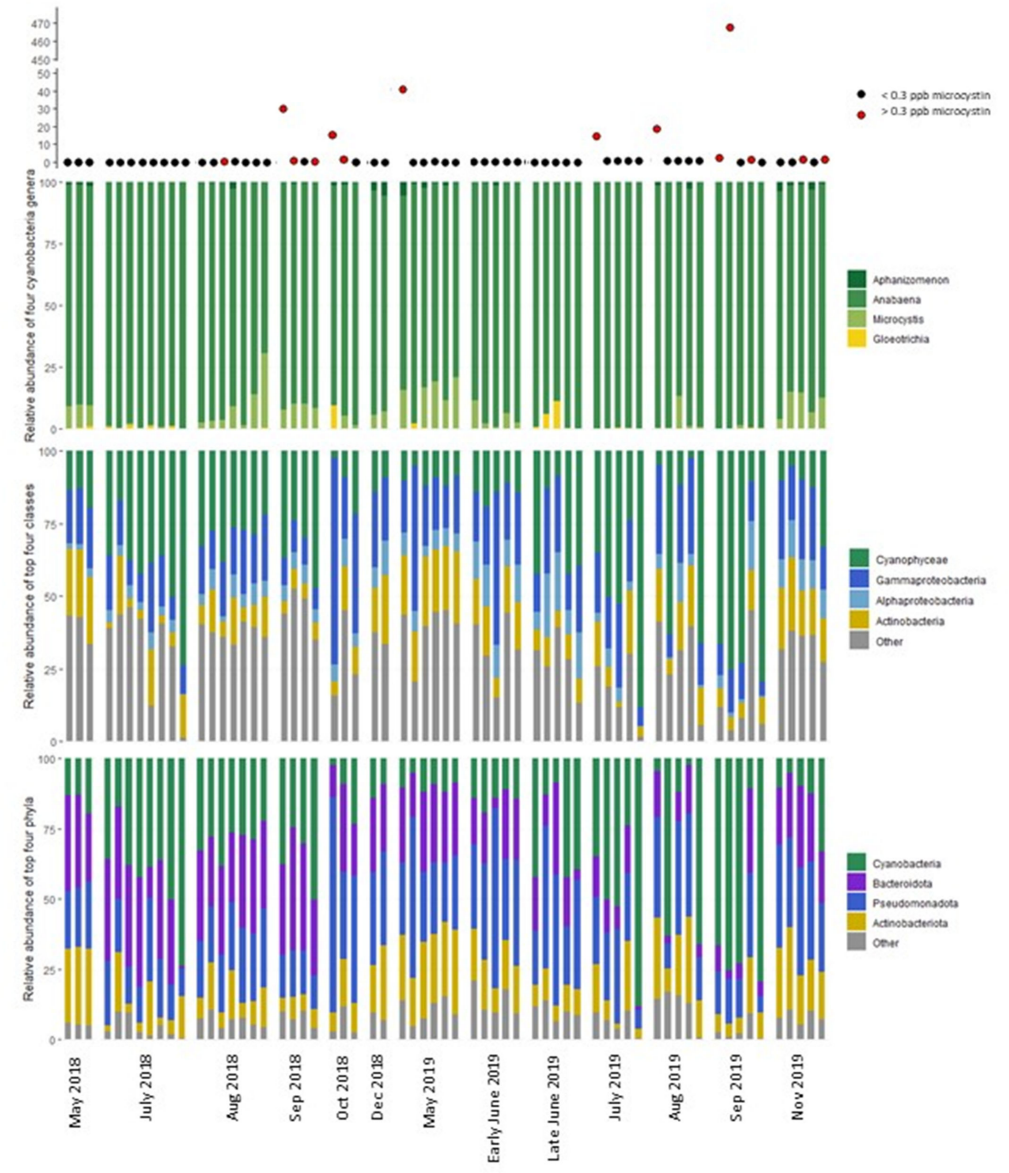
Coarse description of microbial community composition in UKL samples across 2018–2019. Microcystin concentration is shown as points at the top, with concentrations ≥0.3 ppb in red. Relative abundances of the four toxin-producing cyanobacteria genera (top bar graph), four most abundant microbial classes (middle bar graph), and four most abundant microbial phyla (bottom bar graph). Samples are ordered by date on the *x*-axis.

The relative abundances of four phyla, Cyanobacteria, Bacteroidota, Pseudomonadota, and Actinobacteria, represented 79%–99% of the 16S rRNA sequences in all UKL samples during 2018–2019 (Fig. 4). Members of the class Cyanophyceae were only ~10% of the microbial community in May and peaked in September 2019 when they were up to 75% of the community before decreasing in the autumn months. The four bloom-forming and potentially microcystin-producing cyanobacteria genera in ULK were *Aphanizomenon*, *Anabaena/Dolichospermum*, *Microcystis*, and *Gloeotrichia. Anabaena/Dolichospermum* sequences were always the dominant Cyanobacteria, contributing 75%–>99% of the sequences in all samples. The relative abundance of *Microcystis* represented 5%–25% of sequences in August through December and May, but *Microcystis* were absent in June and July.

Cell morphological characteristics of *Aphanizomenon*, which is the dominant Cyanobacteria during the mid-summer in UKL (56), were commonly observed in UKL samples inspected by light microscopy (Fig. S3). Nevertheless, few sequences were placed within *Aphanizomenon* and instead sequences often grouped with representatives of *Anabaena* sp. strain 90, *Dolichospermum circinate* strain ACBU02, and *Anabaena* sp. strain WA 102. 16S rRNA-based phylogenies are so far unable to resolve *Aphanizomenon* and *Anabaena/Dolichospermum* (57). For example, the addition of metagenomic data (with morphological validation) from 16 *Aphanizomenon*, *Anabaena*, and *Dolichospermum* strains collected in the Pacific Northwest of the USA to collections of cyanobacterial genomes used in previous phylogenetic analyses still placed some strains, such as an *Anabaena* strain collected from Washington state, within *Aphanizomenon* (58).

### Microcystin toxin prediction using the volatilome

Elastic net is a regression method that uses regularization to select the input variables that are important for the prediction. We developed elastic net regularized regression models using the UKL volatilome with outputs that were either linearly predictive of microcystin concentration (linear models) or predictive of microcystin concentration ≥0.3 ppb (logistic models) to facilitate different water management approaches (Table 2). Linear model M1 and logistic model M2 were developed using only the 229 *m/z +* 1 values. Linear model M7 and logistic model M8 were developed using the 229 *m/z +* 1 values and “low-cost” environmental parameters (e.g., buoy data such as temperature, pH, and conductivity, which are rapidly retrieved by current technologies) (Table 1). Across the four elastic net models, variable selection identified 24 of the 229 unique *m/z +* 1 as being important to predicting microcystin contamination (Table 3), and their relative concentrations are shown in Fig. 2. Nine *m/z +* 1 values were selected in two elastic net models, and four *m/z +* 1 values (151.119, 157.157, 199.189, and 203.185) were selected in three elastic net models (Table 3).

**TABLE 2:** Models developed for the prediction of microcystin contamination

Model number	Model type	Input variables	Output type
M1	Linear elastic net	VOCs	Continuous
M2	Logistic elastic net	VOCs	Binary
M3	Linear regression	Low-cost environmental parameters	Continuous
M4	Logistic regression	Low-cost environmental parameters	Binary
M5	Linear regression	Low + high-cost environmental parameters	Continuous
M6	Logistic regression	Low + high-cost environmental parameters	Binary
M7	Linear elastic net	VOCs + low-cost environmental parameters	Continuous
M8	Logistic elastic net	VOCs + low-cost environmental parameters	Binary

**TABLE 3:** *m/z +* 1 values identified in models predicting microcystin contamination^*a*^

*m/z* + 1 value	Peak variance	Chemical	Shift (*m*/*z*)	M1	M2	M7	M8
35.042	0.009346	(CH_4_O)H^+ a^	0.004				x
*80.045*	0.02135	(C_5_H_5_N)H^+ ac^ (C_3_H_8_S)H^+ a^ (C_6_H_5_)H^+ a^ (C_4_H_3_N_2_)H^+ b^	−0.004 0.004 −0.008 0.008	C+		C−	
83.055	0.022152	(C_4_H_6_N_2_)H^+ ac^ (C_4_H_4_N_2_)H^+ a^ (C_5_H_6_O)H^+ ac^ (C_3_H_4_N_3_)H^+ b^	−0.005 0.004 0.006 0.007		C+		
83.078	0.022158	(C_6_H_8_)H^+ a^ (C_6_H_10_)H^+ ac^ (C_5_H_8_N)H^+ b^	0.001 −0.008 0.005		C+		
98.040	0.026149	(C_5_H_4_FN)H^+ c^ (C_4_H_4_N_2_O)H^+ a^ (C_3_H_3_N_3_O)H^+ ac^ (C_5_H_5_O_2_)H^+ b^ (C_4_H_5_N_2_O)H^+ b^	0.000 −0.003 0.005 0.003 −0.008			C+	
103.070	0.027491	(C_5_H_10_O_2_)H^+ ac^ (C_5_H_8_O_2_)H^+ a^ (C_3_H_8_N_3_O)H^+ b^ (C_4_H_8_NO_2_)H^+ b^	−0.005 0.006 −0.005 0.007		C+		
111.102	0.029633	(C_7_H_11_N)H^+ a^ (C_8_H_12_)H^+ a^ (C_7_H_12_N)H^+ b^	0.002 −0.006 −0.003			C−	C+
137.129	0.036575	(C_10_H_16_)H^+ acd^ (C_10_H_14_)H^+ a^	−0.003 0.005			C−	
138.131	0.036842	(C_9_H_15_N)H^+ ac^ (C_10_H_16_)H^+ a^	0.003 −0.005			C+	
*148.073*	0.039494	(C_7_H_12_CIN)H^+ a^ (C_9_H_9_NO)H^+ c^ (C_9_H_7_NO)H^+ a^ (C_5_H_10_N_2_O_3_)H^+ a^ (C_6_H_11_O_4_)H^+ b^ (C_7_H_9_N_4_)H^+ b^ (C_2_H_7_N_6_O_2_)H^+ b^ (C_6_H_12_N_2_Cl)H^+ b^ (C_5_H_12_N_2_OP)H^+ b^ (C_4_H_11_N_4_S)H^+ b^ (C_5_H_11_N_2_OS)H^+ b^ (C_7_H_12_OCl)H^+ b^	0.003 −0.003 0.006 −0.007 −0.001 −0.002 0.002 −0.004 −0.004 −0.005 0.006 0.008	C+		C−	
149.117	0.039772	(C_7_H_16_O_3_)H^+ c^ (C_6_H_14_N_2_O_2_)H^+ a^ (C_8_H_18_S)H^+ a^ (C_10_H_13_N)H^+ a^ (C_5_H_14_N_3_O_2_)H^+ b^ (C_10_H_14_N)H^+ b^	0.000 0.000 0.001 0.002 0.001 −0.003	C−		C+	
*151.119*	0.040306	(C_10_H_14_O)H^+ c^ (C_9_H_14_N_2_)H^+ c^ (C_9_H_12_N_2_)H^+ a^ (C_8_H_12_N_3_)H^+ b^	0.007 −0.004 0.005 0.008	C+	C−	C+	
153.095	0.040833	(C_9_H_12_O_2_)H^+ c^ (C_4_H_14_N_3_OP)H^+ a^ (C_7_H_10_N_3_O)H^+ b^ (C_6_H_10_N_5_)H^+ b^	0.004 −0.003 0.005 −0.006			C−	
157.157	0.041917	(C_10_H_20_O)H^+ c^ (C_9_H_18_N_2_)H^+ a^ (C_8_H_18_N_3_)H^+ b^	−0.002 −0.004 −0.001	C−		C−	C−
169.113	0.045106	(C_10_H_16_S)H^+ ac^ (C_9_H_16_NO_2_)H^+ b^ (C_5_H_10_N_7_)H^+ b^	0.008 0.003 0.005			C−	
171.171	0.045655	(C_11_H_22_O)H^+ c^ (C_10_H_20_N_2_)H^+ a^ (C_9_H_20_N_3_)H^+ b^	−0.003 −0.006 −0.003	C+		C−	
175.159	0.046718	(C_10_H_22_S)H^+ c^ (C_9_H_20_NO_2_)H^+ b^ (C_5_H_16_N_7_)H^+ b^	0.008 0.002 0.004	C−		C−	
185.185	0.049392	(C_12_H_24_O)H^+d^ (C_10_H_22_N_3_)H^+ b^ (C_11_H_22_NO)H^+ b^	−0.004 −0.004 0.007	C−		C+	
*189.151*	0.05045	(C_14_H_18_)H^+ a^ (C_13_H_18_N)H^+ b^ (C_8_H_18_N_3_O_2_)H^+ b^ (C_10_H_22_NS)H^+ b^ (C_4_H_14_N_9_)H^+ b^ (C_7_H_18_N_5_O)H^+ b^	−0.004 −0.001 0.003 −0.004 0.006 −0.008			C−	
193.153	0.051517	(C_11_H_17_N_3_)H^+ a^ (C_13_H_18_O)H^+ a^ (C_6_H_18_N_5_O_2_)H^+ b^ (C_11_H_18_N_3_)H^+ b^ (C_12_H_18_N_O_)H^+ b^ (C_6_H_19_N_5_P)H^+ b^	0.000 0.003 −0.001 −0.005 0.006 0.007	C−		C−	
199.189	0.053128	(C_13_H_26_O)H^+ e^ (C_12_H_24_NO)H^+ b^	N/A −0.005		C+	C+	C+
*203.185*	0.054193	(C_7_H_20_N_7_)H^+ b^ (C_11_H_24_NO_2_)H^+ b^ (C_11_H_25_NP)H^+ b^ (C_15_H_22_)H^+ c^	−0.001 −0.004 0.005 0.006	C+	C+	C+	
233.959	0.062401	Many compounds			C+		
*332.868*	0.088782	Many compounds			C+		C+

^*a*^ C+” indicates that the *m/z +* 1 value was retained in the model with a positive coefficient, and “C−” indicates a negative coefficient. Shift is the difference between the chemical’s actual mass and detected mass. Chemical identifications were made using the Ionicon PTR viewer integrated database (“a” superscript), the PTR viewer calculated formulas (“b” superscript), GLOVOC database (“c” superscript), previously published PTR-MS research (“d” superscript), or relationships to other identified *m/z +* 1 values (“e”). Italicized *m/z +* 1 values were also important in predicting cyanobacterial relative abundance (Fig. 6).

**Fig 5:**
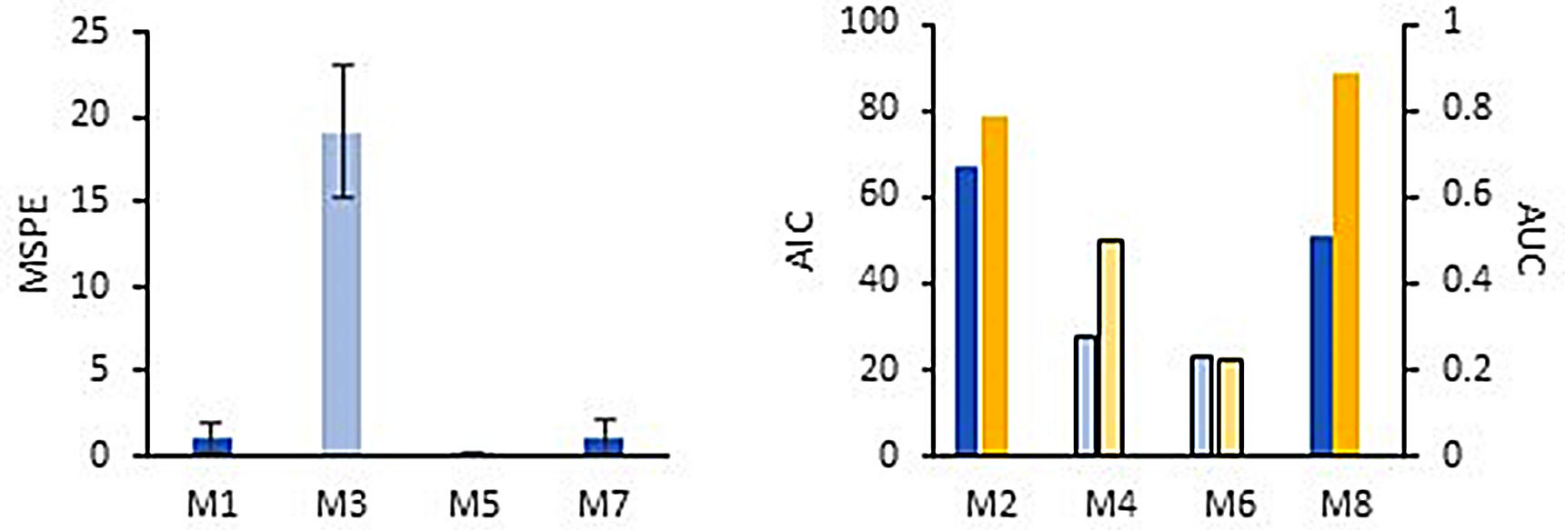
Statistical performances of linear models (left) and logistic models (right) predicting microcystin contamination. VOC-based M1, M2, M7, M8 (dark blue) and environmental parameter-based M3, M4, M5, M6 (light colors). AIC, blue bars; AUC, yellow bars. Error bars are SD.

Four additional regression models based on the “low-cost” environmental parameters (M3, M4) or the full collection of environmental parameters (“low + high cost”, M5, M6) were developed to compare against the performance of the VOC-based elastic net models (Table 2). Similar to previous studies (59), “low-cost” linear M3 was weakly predictive of microcystin concentration (MSPE, 19.1) and retained only pH and chlorophyll (Table S1). POC, PON, and AMM strongly boosted the predictive power of linear M5 (MSPE < 1). Neither logistic “low-cost” M4 nor “low + high cost” M6 were able to discriminate whether samples contained microcystin ≥0.3 ppb with greater than 50% accuracy (Fig. 5; Table S2; Fig. S4).

All of the VOC-based models outperformed “low-cost” comparator models to predict microcystin in UKL (Fig. 5). Addition of “low-cost” environmental parameters to the training data did not improve VOC-based model performance (Fig. 5), and except for “month” in M8, were not retained in the final equations (Tables S1 and S2). The high Akaike Information Criterion (AIC) in logistic M2 and M8 are partly attributable to the relatively high number of selected variables (8 and 18, respectively) and were strongly balanced by area under the receiver operating characteristic curve (AUC) values that were 0.78 and 0.88 compared to 0.50 (no better than chance) for M4 and only 0.22 for M6 (Fig. 5; Fig. S4).

VOCs were effective predictors of microcystin in UKL. Our ability to rapidly measure volatile metabolites in water samples requiring no pre-processing (5-minute PTR-MS measurement of raw water samples) provides a unique platform to explore relationships between the volatilome and ecosystem health and the potential for VOCs to be leveraged in cyanotoxin monitoring. Low volatility of toxins, including microcystin, makes their direct detection by PTR-MS unfeasible. Direct toxin measurement by ELISA or mass spectrometry is the current gold standard for monitoring but can, at times, become too expensive for frequent and widespread application across sensitive waterways dependent on timely public health advisories (19). The metabolome is increasingly used to evaluate human health (60 – 62) and ecosystem status, such as shifts in soil microbial ecology (63). Similarly, the success of the volatilome to provide information about microcystin presence and concentration suggests that unique collections of VOCs in UKL are produced depending on cell physiology and community composition.

### Predicting microbial community composition using the volatilome

**Fig 6:**
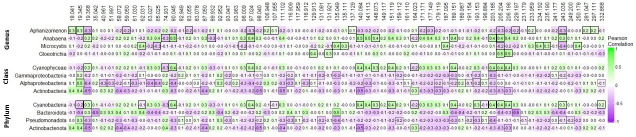
Pearson correlations between the relative abundances of the four toxin-producing cyanobacterial genera (top), four most abundant microbial classes (middle), or four most abundant microbial phyla (bottom) in UKL samples and the associated *m/z +* 1 values identified in elastic net models (shown at top). Outlined boxes are *m/z +* 1 values identified in the model predicting the relative abundance of the taxonomic group in each row. Boxes with a solid outline indicate *m/z +* 1 values in models predicting relative abundances of cyanobacteria genera, class, or phylum. Note that some *m/z +* 1 values predictive of cyanobacteria genera relative abundances are also predictive of Cyanophyceae and Cyanobateria relative abundances. Boxes with a dotted outline indicate *m/z +* 1 values in models predicting the relative abundance of other taxonomic classes or phyla. Pearson’s *r* value of 1 (green) indicates a positive correlation, and a value of −1 (purple) indicates a negative correlation.

Elastic net models were also developed using the relative abundances of the four most abundant phyla, classes, and toxin-producing cyanobacteria genera as dependent variables and the 229 *m/z* + 1 values as independent variables. The 12 resulting models selected a total of 71 *m/z +* 1 values (Table S3). All 12 elastic net models performed well, yielding mean squared prediction errors (MSPEs) that were 0.75–1.02 and SDs that were 0.08–0.54 (Fig. S5). The *m/z +* 1 value 205.204 was an important predictor of the relative abundance of Cyanobacteria phylum, Cyanophyceae class, and all four Cyanobacteria genera (Fig. 6). Eleven of the 18 *m/z +* 1 values predictive of the Cyanobacteria phylum relative abundance were also predictive of Cyanophyceae relative abundance and 14 were predictive of the relative abundance of at least one of the Cyanobacteria genera. Similarly, seven of the eight *m/z +* 1 values predictive of Actinobacteriota relative abundance were predictive of Actinobacteria (class) relative abundance (Fig. 6). Six *m/z +* 1 values identified in models predicting microcystin concentration were also identified in models predicting the relative abundances of Cyanobacteria genera (Table 3).

Elastic net regularized regression yielded a collection of VOC-based models that were highly effective at predicting the relative abundance of key cyanobacteria, including *Microcystis*, which is thought to be the primary source of microcystin in UKL. The success of these models is likely a consequence of seasonal changes in the microbial community composition and taxonomic and physiological differences leading to the collection of VOCs released (32, 64 – 66). We do not know if the VOCs identified here would also be detected in cultures of the different cyanotoxin-producing cyanobacteria. Fundamental differences in metabolism between strains in culture collections could result in unique volatilomes, and the absence of VOCs observed in this study in cultures may be a consequence of *in situ* conditions rather than a VOC-strain association. Furthermore, the complex interactions between cyanobacteria and ecosystem processes leading to toxin production remain enigmatic and challenging to replicate in the laboratory. Nevertheless, the subsets of VOCs identified using elastic net revealed *m/z +* 1 values that were maintained through coarse and finer-grained taxonomic groups. These results indicate systematic relationships between volatilomes and microbial taxa in UKL. We are unaware of studies that have leveraged the metabolome to describe microbial community composition; however, neural networks and linear regression approaches are being used to integrate metabolomic, metagenomic, and taxonomic data (67 – 70). In our study, elastic net regularized regression applied to volatilomes yielded models that were strongly predictive of the cyanotoxin and microbial community composition.

Selected *m/z +* 1 values in our models suggest that those compounds mediate interactions between cyanobacteria, microcystin, and the environment. For example, a sesquiterpene, *m/z +* 1 203.185, was retained with positive coefficients by three models predicting microcystin and in models predicting relative abundances of Phylum Cyanobacteria, Cyanophyceae, and *Anabaena*. Sesquiterpene synthases are present in *Anabaena* species (71), and the recurrence of *m/z +* 1 203.185 in our models is consistent with the abundance of *Anabaena* in UKL and the release of sesquiterpenes and microcystin during cyanobacterial senescence (72).

β-ionone was assigned to *m/z +* 1 193.153 based on its known PTR-ToF-MS target mass (73). *m/z +* 1 193.153 was retained with negative coefficients in M1 and M7 predicting microcystin and three models predicting the relative abundance of non-cyanobacterial taxonomic groups. *m/z +* 1 193.153 was positively correlated with phylum Cyanobacteria, Cyanophyceae, and *Anabaena* (Fig. 6). β-ionone and other norcarotenoids are products of carotenoid oxidation in various cyanobacteria during photo-oxidative stress, including *Anabaena*, *Aphanizomenon*, *and Microcystis*, and inhibit photosystem II in *Microcystis* (38, 74 – 77). Oxidative stress in UKL may have induced the production of β-ionone in cyanobacteria (78, 79), thereby decreasing *Microcystis* abundance and microcystin production. Non-toxic *Microcystis* strains employ peroxidases in response to oxidative stress, but toxic *Microcystis* strains may produce microcystin to combat mild, chronic oxidative stress (80). The different pathways employed by cyanobacteria to tolerate oxidative stress point to β-ionone as a potentially important compound that mediates interactions within the cyanobacterial community, including microcystin production. β-ionone is also a taste-odor compound in potable freshwater sources (74) that can be rapidly identified using our approach.

*m/z +* 1 137.129 is likely limonene with the molecular formula (C_10_H_14_)H^+^. Limonene is a monoterpene produced by planktonic and benthic cyanobacteria (81). Other compounds with the same *m/z +* 1 value reported in PTR-MS databases include pinene and linalool, but neither of these terpenes are produced by wild-type cyanobacteria (82, 83). *m/z +* 1 137.129 was retained with a negative coefficient in M7 and a positive coefficient in the model predicting the relative abundance of *Aphanizomenon. m/z +* 1 137.129 was also negatively correlated with *Microcystis* and *Gloeotrichia* (Fig. 6). Limonene can inhibit photosynthesis (37, 84) and lyse *Microcystis aeruginosa* (85), suggesting that limonene produced by *Aphanizomenon* was associated with lower *Microcystis* abundance and perhaps consequently, lower microcystin concentrations.

The *m/z +* 1 values 157.157, 171.171, and 185.185 were selected in M1, M7, and M8, respectively, and differ by 14.014 mass units, suggesting these VOCs are products of sequential demethylation activity. A fourth, *m/z +* 1, 199.189, is 14.004 mass units greater than 185.185 and was retained in M2, M7, and M8 with positive coefficients. The lowest *m/z +* 1 value in this series, 157.157, was retained with negative coefficients. Chemical formulas for these *m/z +* 1 values include C10–C13 saturated fatty aldehydes (SFAs), decanal, undecanal, dodecanal (86), and tridecanal (Table 3). Tridecanal is a key marker for Cyanophyceae (87) but is not yet present in PTR-MS chemical databases and has not been reported in PTR-MS-based research. Nevertheless, the longer chained SFAs (C12 and possibly C13) appear to be upregulated in concert with microcystin production.

**Fig 7:**
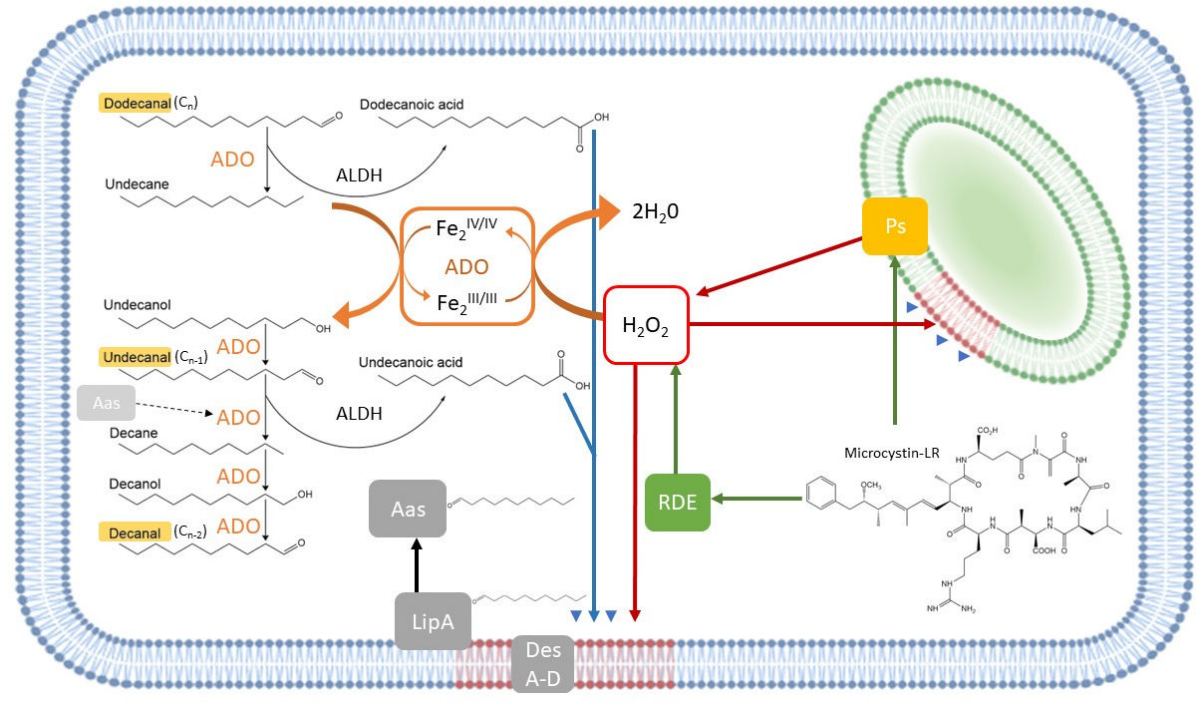
The role of the saturated fatty aldehyde (SFA) oxidation pathway in membrane lipid repair and depletion of reactive oxygen species. SFAs putatively identified by elastic net models predicting microcystin concentration are in yellow (*m/z* + 1 values 157.157, 171.171, and 185.185). SFAs produced by aldehyde-deformylating oxygenase (ADO) are metabolized by aldehyde dehydrogenase (ALDH) producing saturated fatty acids. Blue arrows and arrowheads represent saturated fatty acids used to repair thylakoid (green) and plasma membrane (gray) lipids damaged by ROS (red), represented here by H_2_O_2_. Photosynthesis (Ps) unavoidably produces ROS. Medium chained alkanes (C10–C12) reduce ROS via ADO activity (see text). Microcystin can protect against ROS (green arrows) by binding to the photosynthetic subunits and by binding to and promoting the production of ROS-degrading enzymes (RDE). Desaturases A-D (DesA-D) unlink glycerol from the fatty acyl moieties in the membrane. The fatty acyl is removed from the membrane by lipolytic enzyme, LipA. Acyl-ACP synthase (Aas) can reattach the fatty acyl to ADO to re-enter the SFA oxidation pathway, represented by a dotted arrow.

The associations between SFAs and microcystin concentration in our elastic net models indicate that the relative abundances of SFAs shift during oxidative stress. SFAs accumulate between the lipid bilayers of cyanobacterial thylakoid and cytoplasmic membranes (50, 88) where they contribute to membrane structure and help fine-tune localization of photosynthetic machinery (50) during temperature and light stress (49, 50, 89). Cyanobacteria use an acyl-acyl-carrier-protein (ACP) reductase/ADO pathway to produce fatty aldehydes of decreasing chain length (C_*n*_, C_*n* − 1_, C_*n* − 2_, … ; Fig. 7). Fatty aldehydes are substrates for aldehyde dehydrogenase (ALDH) yielding fatty acids that can be used to repair membrane lipids damaged by ROS (i.e., hydrogen peroxide, H_2_O_2_) produced during photosynthesis. ADO, like other diiron oxygenases, appears to be a powerful oxidizing enzyme with a wide substrate range (51). The alkane products of NADH-dependent ADO activity on fatty aldehydes can also serve as electron donors to reduce ROS. This latter reaction is primed by the generation of a Fe^IV^-Fe^IV^ diiron center in ADO by H_2_O_2_ and alkane oxidation returns the diiron center to the Fe^III^-Fe^III^ state (90). However, alkane-dependent H_2_O_2_ reduction could also lead to OH^•^ accumulation causing a deleterious cycle of cell damage. During high light stress, the fatty acid and alkane metabolites of the ADO pathway would be rapidly depleted requiring larger pools of longer chain-length fatty acids to maintain ongoing lipid repair and H_2_O_2_ destruction. The genes encoding ADO and ALDH were upregulated in the model cyanobacterium, *Synechocystis* sp. PCC6803 during high light and oxidative stress (91, 92). As the cell’s capacity to repair systems damaged by ROS becomes overwhelmed, longer chained SFAs may accumulate, making them useful targets for microcystin detection by PTR-MS.

One current prevailing hypothesis for a biological role for microcystin in cyanobacterial cells posits that it protects photosystems and peroxidases against oxidative damage (80, 93 – 95). Increased cyanobacterial dependence on the ADO pathway during oxidative stress is consistent with the mechanistic view that ROS can rapidly accumulate to algicidal concentrations even in the presence of microcystin. Although the *m/z +* 1 values retained in our microcystin models and assigned here to SFAs need to be independently verified using standards or other mass spectrometry approaches (e.g., gas chromatography-mass spectrometry), selection of this collection of related *m/z +* 1 values in multiple elastic net models predicting microcystin concentration suggests SFAs’ ecological and biochemical interactions with microcystin production (Fig. 7).

The use of VOCs to evaluate microcystin and microbial composition in UKL is time-efficient and could be streamlined or even automated to inform agencies and water managers within a day. The volatilome in water samples was collected directly by PTR-TOF-MS without the need for pre-processing or sorption onto resins. Our models were designed to determine total microcystin concentration and cannot at this time evaluate toxicity, which would require knowledge of the abundances of specific microcystin congeners (96, 97). Evaluation of the volatilome is a holistic and indirect measurement of the ecosystem. Many of the *m/z +* 1 values identified in our elastic net models provide valuable targets for future study of their roles in cell- to ecosystem-level processes.

### Conclusion

The increasing frequency and severity of toxic cyanoHABs in waterways makes new, cost-effective monitoring strategies an urgent task. The ideal monitoring approach would yield information about cyanotoxin identity and concentration, cyanobacterial abundances, and ecosystem health. The VOCs produced in Upper Klamath Lake, OR, provided information about the integrated growth environment and were leveraged using regularized regression to determine microcystin concentration and microbial community composition in UKL water samples. Specific VOCs, including SFAs, may be the smoking gun needed to quickly detect toxin production in freshwater lakes.

Cyanotoxins can now be detected in many waterways that were thought to be pristine, suggesting that the combination of ongoing human activities and climate change is shifting many waterways toward ecological tipping points where HABs and cyanotoxin contamination are reliable annual events. Application of volatilomes and complex data analysis shows their potential for the guidance of water treatment for taste-odor compounds in drinking water, monitoring of toxic and non-toxic cyanoHABs, and novel discovery of ecological interactions leading to toxin production *in situ*. An important next step is to determine whether the identified *m/z +* 1 values in our models emerge in samples from other lakes experiencing toxic cyanoHAB events. Because water manager actions are predicated on sensitive and timely detection of cyanotoxins and their bacterial producers, future research that harnesses volatilomes in conjunction with other accessible complex data, including real-time buoy and satellite monitoring, to track and predict cyanoHAB trajectories before, during, and after toxic HAB events, is warranted to limit public exposures and economic hardship.

## MATERIALS AND METHODS

### Water sample collection

Water samples were collected from three sites on Upper Klamath Lake and one site on its northern arm, Agency Lake, during the months of May–December in 2018 and 2019. Sampling sites were NAL, an agricultural-dominated terrain on the northeastern shore of Agency Lake, two wildlife and recreational areas near the peninsula at Eagle Point in UKL (EPP) and western shore of UKL at Howard Bay (WBR), and a residential area near the dam at the southern end of the lake (PEL) (Fig. 1). Additional samples were collected from canals that drain from UKL for agricultural irrigation (NCA, ADY, MER, and LRI; Fig. 1). Samples were collected by pole from the surface about 2 m from the shore or canal line. Samples for all analyses (VOCs, anions, pigments, particulate C and N, community composition) excepting microcystin concentration were collected in autoclaved 1-L polycarbonate bottles with limited to no headspace. Samples were transported in a cooler to Oregon State University, Corvallis, OR. VOCs were measured and microscopic analysis was conducted within 24 hours of collection. Samples for POC, PON, chlorophyll, and 16S rRNA sequencing were filtered and frozen within 24 hours of collection. Samples for microcystin concentration were collected on site in autoclaved 10-mL glass vials and frozen on arrival at Oregon State University (−20°C) for later analysis. Samples for ion measurement were frozen for later analysis at Oregon State Universities Freshwater IIW Collaboratory.

### Environmental parameters

Temperature, pH, and conductivity were measured on site using an Extech pH/temperature meter (Nashua, NH) and YSI 30 Conductivity meter (Yellow Springs, OH), respectively. The anions bromide, fluoride, chloride, nitrite, nitrate, phosphate, and sulfate were measured with a Dionex ICS-1500 Ion Chromatograph Autosampler (Sunnyvale, CA). Data for bromide and fluoride are not shown because only two samples yielded data above the detection limits. Ammonium was measured by UV-Vis spectroscopy after three freeze-thaw cycles (98). POC and PON were determined from three volumes (3–110 mL) filtered onto pre-combusted GF/F filters to create a linear regression, and frozen until analysis by Exeter Analytical EA1 elemental analyzer (99) (Coventry, England). Non-particulate C and N were determined from sample filtrate and subtracted from the filtered samples. The median sample volume of filtrate was re-filtered onto a fourth GF/F filter, frozen, and analyzed with the sample filters. Chlorophyll concentration was measured in triplicate using 2–100 mL of sample filtered onto 25-mm GF/F filters (until green was observed on the filter) and extracted for 24–48 hours at −20°C in 90% acetone. Extract absorption was measured by spectrophotometer (Shimadzu, Kyoto, Japan) and calculated using the equation for cyanobacteria from Ritchie (2006) (100). Microcystin concentrations were measured using Eurofins Abraxis Inc. Microcystins/Nodularins (ADDA) ELISA Kit (Product No. 520011).

### Detection of VOCs

Triplicate 100-mL sub-samples were transferred to custom-made 200-mL polycarbonate dynamic stripping chambers with sintered glass frits (2–2.5 µm) at the bases (32). Chambers were placed in an incubator at the sample collection temperature. Samples were stripped of VOCs by flowing synthetic air through a hydrocarbon trap, then a flow controller (Sierra Instruments) set to 50 sccm, and then through the glass frits into the samples. The carrier air with the stripped VOCs was directed into the PTR-TOF-MS (Ionicon, Austria) where the primary ion (H_3_O^+^) causes a proton transfer reaction, or soft ionization event, to VOCs having higher proton affinities than 691 kJ/mol, which is the proton affinity of water. VOCs in the mass range of 18–363 a.m.u. were detected at their molecular masses plus one (*m/z +* 1). Data were collected over 5 minutes. The conditions of the drift tube were 2.1 mbar, 80°C, and 500 V with an E/N value of 125 Td.

### VOC data processing

PTR-TOF-MS raw peak data were processed using PTRwid (101). The resulting output yielded tables giving each integrated *m/z +* 1 peak signal that incorporated a correction for overlapping peaks. PTRwid yields a unified mass list of all *m/z +* 1 values detected in all lake and canal samples. Known contaminants and internal standards were removed from the list prior to subsequent data processing (Table S4). The first 2.5 minutes of data were removed to account for contaminating air in the tubing and headspace of the stripping chambers. The remaining data were integrated over 2.5–5 minutes. Differences in concentrations of *m/z +* 1 values and Wald’s test derived *P*-values were determined using the R package *DESeq2* (102), and Benjamini-Hochberg corrected *P*-values of *m/z +* 1 values were determined using the EnhancedVolcano package (102). Chemical formulas were assigned using Ionicon PTR Viewer software and the Ionicon integrated database (PTR Viewer software version 3.3), PTR Viewer calculated values (version 3.4.2) or GLOVOCs database for PTR-MS (103) (update November 16, 2020). Some chemical formulas were assigned based on published PTR-MS research on those compounds. The maximum mass shift (difference between the actual mass value and the detected mass value) allowed for the compound assignment was 0.007 a.m.u. as determined by PTR-TOF-MS calculated RMSE.

### VOC-based elastic net models predicting microcystin concentration

Two elastic net model types were developed: (i) linear models that predict the continuous outcome of microcystin concentration (M1 and M7) and (ii) logistical models that predict the dichotomous outcome of whether microcystin concentration ≥0.3 ppb (M2 and M8). The logistical models were trained with a binary output that designated a sample as toxic if the microcystin concentration was at or above the 0.3 ppb threshold. M1 and M2 utilized only *m/z +* 1 values and were trained using the *glmnet* function in R software (version 4.1.0) on 95% of the samples (total *n* = 70). M7 and M8 utilized *m/z +* 1 values and “low-cost” environmental variables (Table 2) and were trained on 95% of all samples for which VOC and “low-cost” environmental data were available (total *n* = 35). Cross-validation with 10- (M7, M8) or 15 (M1, M2)-folds was used to determine the values of the tuning parameters (Table S2), and hence the strength of regularization in M1, M2, M7, and M8. MSPE and their SD in M1 and M7 were calculated based on the model with the tuning parameter set to the value yielding the minimum mean cross-validated error. AUC and AIC for M2 and M8 were averaged from 10 random iterations of each model. The *m/z +* 1 values in the final models were selected using the full data sets. The *m/z +* 1 values retained by the elastic net models were refit using the lm function in R to yield the coefficients in each final model (Table S2). A lower MSPE, lower AIC, and higher AUC are indicative of a preferred model.

### “Low cost” and “low + high-cost” regression models predicting microcystin concentration

Four base models were developed to predict microcystin concentration using only environmental parameters (Table 2). Outlier removal and bidirectional stepwise elimination were implemented using the *MASS* package in R to select the environmental parameters. Microcystin concentration in M3 was modeled by multiple linear regression using the month of collection, the collection site, and “low-cost” environmental parameters. Multiple linear regression model, M5, was based on the month of collection, collection site, and both “low-cost” and “high-cost” environmental parameters (Table 3). Two logistic models were developed based on “low-cost” and “low + high-cost” environmental parameters (M4 and M6, respectively) to predict microcystin concentration ≥0.3 ppb. The predictive performance of these linear regression base models was evaluated using the *glmnet* (104, 105) package with lambda and alpha values set to zero.

### DNA extraction and sequencing

Samples (10–150 mL) were filtered onto 0.2-µm polycarbonate filters and stored at −20°C until DNA extraction using phenol:choloroform:iso-amyl 25:24:1. DNA quality and quantity were determined by NanoDrop 1000^104^. The V1–V2 region of the 16S rRNA gene was amplified using 27F (5′-AGAAGAGTTTGATCNTGGCTCAG-3′) and 338 RPL (5′-CWGCCWCCCGTAGGWGT-3′) primers with overhang adaptors according to the Illumina Inc. standard 16S sequencing library preparation protocol. Libraries were created using dual indices and Illumina sequencing adapters with a Nextera XT Index Kit (Illumina Inc.), then pooled in equimolar concentrations and sequenced using Illumina MiSeq (2× 250 PE) in two batches: 54 samples were sequenced at the Center for Quantitative Life Sciences (Oregon State University, Oregon), and 16 samples were sequenced at Molecular Research DNA-RNA Laboratory (Shallowater, TX) (106).

### 16S rRNA gene amplicon analysis

The 27F (20 bp) and 338RPL (18 bp) primers were removed using CutAdapt, then DADA version 1.2 R package (version 3.6.1), using the SILVA database train version 138, quality-filtered, dereplicated, merged, constructed an ASV table, removed chimeras, and taxonomically assigned the sample reads through the dada2 package (106). Taxonomic assignment through dada2 was used for phylum and class classification. Taxonomic assignment of 16S rRNA sequences within the Cyanobacteria genera was done using Cydrasil and its maximum-likelihood phylogenetic tree constructed of 1,327 Cyanobacteria reference sequences (107). Sequences were aligned using reference alignments constructed using PaPaRa version 2.0, and the alignments were placed using EPA-ng (107). The placements were visualized via the Interactive Tree of Life (iTOL) version 6.5.4, and taxonomy was hand-assigned (Fig. S6) (108). The dominant cyanobacteria were also confirmed by visual identification using light microscopy and morphological characteristics (Fig. S3).

### VOC-based models of microbial community composition

Relative abundances of the four most abundant microbial phyla, classes, and cyanobacterial genera (*Anabaena*, *Aphanizomenon*, *Gloeotrichia*, and *Microcystis*) were determined using the R package phyloseq (109). A total of 12 VOC-based linear regression models were developed to predict microbial relative abundances using elastic net modeling. Models were trained using the *glmnet* function in R (version 4.1.0) on 95% of the samples (total *n* = 70). Cross-validation with 15-folds was used to determine the value of the tuning parameter (Table S3) and hence th strength of regularization. MSPE and their SDs were calculated from the final model with the tuning parameter set to the value yielding the minimum mean cross-validated error. The *m/z +* 1 values in the final models were selected using the full data set. The *m/z +* 1 values retained by the elastic net models were refit using the lm function in R to yield the coefficients in each final model (Table S3). The R package *ComplexHeatMap* (110) was used to create Fig. 2 and 6, and Fig. S2, and the R package *EnhancedVolcano* (111) was used to create Fig. 3.

## Data Availability

The authors declare that all data supporting the results of this study are available within the article and corresponding supplemental material. 16S rRNA sequencing data have been deposited to National Center for Biotechnology Information (NCBI accession no. PRJNA922214), and the model code is available on Open Science Framework.

## SUPPLEMENTAL MATERIAL

Table S3 msystems.00379-23-s0001.csv MSPE, SD, intercept, alpha value, and coefficients for *m/z*+*1* values that were retained in the elastic net models predicting bacterial relative abundance.

Supplemental figures and tables msystems.00379-23-s0002.docx Tables S1, S2, and S4; Figures S1-S6.

ASM does not own the copyrights to Supplemental Material that may be linked to, or accessed through, an article. The authors have granted ASM a non-exclusive, world-wide license to publish the Supplemental Material files. Please contact the corresponding author directly for reuse.
