# Supplementary material for: The volatilome reveals microcystin concentration, microbial composition, and oxidative stress in a critical Oregon freshwater lake
Source: mSystems. 2023 Aug 17;8(5):e00379-23. doi: 10.1128/msystems.00379-23 (PMC10654074; doi:10.1128/msystems.00379-23)
Supplement: Supplemental figures and tables — Tables S1, S2, and S4; Figures S1-S6. [file msystems.00379-23-s0002.docx]

Supplementary information

Table S1. Environmental parameters identified in linear and logistic regression models predicting microcystin contamination using bidirectional stepwise elimination (M3-M6). An ‘x’ indicates the parameter was selected: parameters identified in two models are shaded light orange, and parameters identified in three models are shaded dark orange. Note that no environmental parameters were selected in M7 and M8.

| **Environmental parameter** | **M3** | **M4** | **M5** | **M6** | **M7** | **M8** |
| --- | --- | --- | --- | --- | --- | --- |
| Chlorophyll | x | x |  |  |  |  |
| pH | x | x |  | x |  |  |
| Temperature |  | x |  |  |  |  |
| Particulate organic carbon |  |  | x | x |  |  |
| Particulate organic nitrogen |  |  | x | x |  |  |
| Sulfate |  |  |  | x |  |  |
| Ammonium |  |  | x | x |  |  |

Table S2. Model equations for prediction of microcystin concentration using VOCs, environmental parameters, or both. Abbreviations for environmental parameters are given in Table 1.

| **Model** | **Equations:** $\hat{\mathbf{Y}}$ **is microcystin concentration in ppb; logit(p) is the log odds of microcystin ≥0.3 ppb** | **Statistics** | **Alpha** |  |
| --- | --- | --- | --- | --- |
| M1^a^ | $\hat{Y}$ = – 11.02 + 4.88(80.045) + 11.22(148.073) -1.35(149.117) + 29.94(151.119) – 40.39(157.157) + 58.46(171.171) – 72.06(175.159) – 10.18(185.185) – 11.21(193.153) + 6.78(203.185) | MSPE=1.03  SD=0.90 | 0.015 |  |
| M2^a^ | logit(p) = -10.17 +0.06(83.055) + 0.01(83.078) + 0.15(103.070) – 0.09(151.119) + 0.05(199.189) + 0.97(203.185) + 3.68(233.959) + 7.25(332.868) | AIC=66.66 AUC=0.78 | 0.8 |  |
| M3^b^ | $\hat{Y}$ = 2.24(logCH) – 3.08(PH) + 52.92 | MSPE=19.10 SD=3.92 | - |  |
| M4^b^ | logit(p) = 0.52(Month)^2^ – 8.57(SiteNAL) – 3.85(SiteWBR) + 1.76(logCH) + 1.94(TEMP) -2.54(PH)– 31.95 | AIC=27.14 AUC=0.50 | - |  |
| M5^c^ | $\hat{Y}$ = 7.47(Month) – 0.50(Month)^2^ + 3.027(POC) – 15.37(PON) + 6.12(AMM) – 25.86 | MSPE = 0.15  SD = 0.05 | - |  |
| M6^c^ | logit(p) = [7.44(Month) – 0.40(Month)^2^ + 1.71(PH) + 0.50(POC) – 2.59(PON) + 0.59(SUL) +3.01(AMM) – 5.08] · 10^5^ | AIC=22.99 AUC=0.22 | - |  |
| M7^a^ | $\hat{Y}$ = – 19.15(80.045) + 48.87(98.040) – 5.25(111.102) -18.39(137.129) + 198.95(138.131) – 20.49(148.073) + 17.80(149.117) + 30.65(151.119) – 8.43(153.095) – 37.61(157.157) – 21.23(169.113) – 28.741(171.171) –423.44(175.159) + 20.01(185.185) – 19.68(189.151) – 43.99(193.153) + 5.69(199.189) + 147.59(203.185) – 32.44 | MSPE = 1.09  SD = 1.02 | 0.008 |  |
| M8^a^ | logit(p) = 0.29(35.042) + 0.09(111.102)-0.07(157.157) + 0.80(199.189) + 13.77(332.868) + 0.14(Month)–11.45 | AIC 50.37 AUC=0.88 | 0.45 |  |

^a^*m/z+1* values selected by the model are given in parentheses

^b^”low-cost” parameters selected by the model are given in parentheses

^c^”low-cost” and “high cost” parameters selected by the model are given in parentheses

Excel Table S3. showing the MSPE, SD, intercept, alpha value, and coefficients for *m/z+1* values that were retained in the elastic net models predicting bacterial relative abundance. *m/z+1* values retained by elastic net models predicting microcystin contamination are highlighted.

Table S4. Table of removed contaminants and isotopic pattern peaks (IPP). Inclusion of IPPs that are highly correlated can negatively impact an elastic net analysis that leans heavily towards the L1-norm (as used in a least absolute shrinkage and selection operator, or LASSO, analysis).

| Mass removed | Type | Notes |
| --- | --- | --- |
| 31.988 | Contaminant | O2+ (ion source) |
| 32.992 | Contaminant | O2H+ (ion source) |
| 33.993 | Contaminant | H2O.H3O+ (hydrate cluster) |
| 37.044 | Contaminant | H2O.H317O+ (hydrate cluster) |
| 38.033 | Contaminant | 3H2O.H3O+ (hydrate cluster) |
| 39.033 | IPP | Mass 38.033 |
| 44.05 | IPP | Mass 43.046 |
| 44.993 | Contaminant | CO2H+ (contaminant peak) |
| 47.022 | Contaminant | NO2+ (ion source contaminant) |
| 51.009 | IPP | Mass 50.014 |
| 55.052 | Contaminant | 15NO2+ (ion source contaminant) |
| 57.036 | Contaminant | 2H2O.H3O+ (hydrate cluster) |
| 57.064 | Contaminant | 2H2O.H318O+ (hydrate cluster) |
| 58.072 | IPP | Mass 57.064 |
| 61.03 | IPP | Mass 60.049 |
| 64.029 | IPP | Mass 63.027 |
| 65.025 | IPP | Mass 63.027 |
| 73.057 | Contaminant | 3H2O.H3O+ (hydrate cluster) |
| 74.036 | IPP | Mass 73.057 |
| 76.023 | IPP | Mass 75.023 |
| 82.067 | IPP | Mass 81.063 |
| 84.077 | IPP | Mass 83.078 |
| 86.07 | IPP | Mass 85.064 |
| 88.051 | IPP | Mass 87.05 |
| 89.052 | IPP | Mass 87.05 |
| 94.073 | IPP | Mass 93.047 |
| 98.04 | IPP | Mass 97.069 |
| 102.087 | IPP | Mass 101.053 |
| 122.102 | IPP | Mass 121.092 |
| 123.963 | Contaminant | Teflon rings (drift tube contaminant) |
| 136.033 | IPP | Mass 135.049 |
| 144.123 | IPP | Mass 143.131 |
| 150.045 | IPP | Mass 149.042 |
| 156.094 | IPP | Mass 155.065 |
| 158.168 | IPP | Mass 157.157 |
| 186.201 | IPP | Mass 185.185 |
| 192.154 | IPP | Mass 191.154 |
| 203.955 | Contaminant | 1,3-diiodobenzene (internal calibrant) |
| 204.959 | Contaminant | 1,3-diiodobenzene (internal calibrant) |
| 205.956 | Contaminant | 1,3-diiodobenzene (internal calibrant) |
| 210.159 | IPP | Mass 209.162 |
| 220.18 | IPP | Mass 219.182 |
| 222.16 | IPP | Mass 221.166 |
| 235.19 | IPP | Mass 234.192 |
| 282.051 | IPP | Mass 281.047 |
| 298.12 | IPP | Mass 297.111 |
| 299.115 | IPP | Mass 297.111 |
| 329.904 | Contaminant | 1,3-diiodobenzene (internal calibrant) |
| 330.931 | Contaminant | 1,3-diiodobenzene (internal calibrant) |
| 331.935 | Contaminant | 1,3-diiodobenzene (internal calibrant) |

Figure S1. Correlation plot of the independent environmental variables calculated using Pearson correlation coefficient.


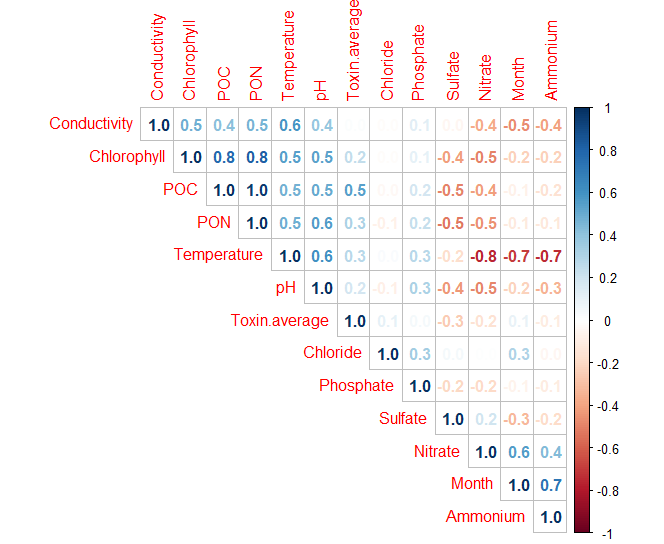


Figure S2. Heatmap of all centered and scaled *m/z+1* values detected in UKL. Samples are listed on the right side of heatmap, and arranged by dendrogram based on Pearson correlation values. *m/z+1* values are listed on the bottom side of heatmap and ordered by mass. The magnitude of correlation for each m/z+1 value to the sample date and site is represented by the color shift, with yellow being a positive correlation and blue a negative correlation.


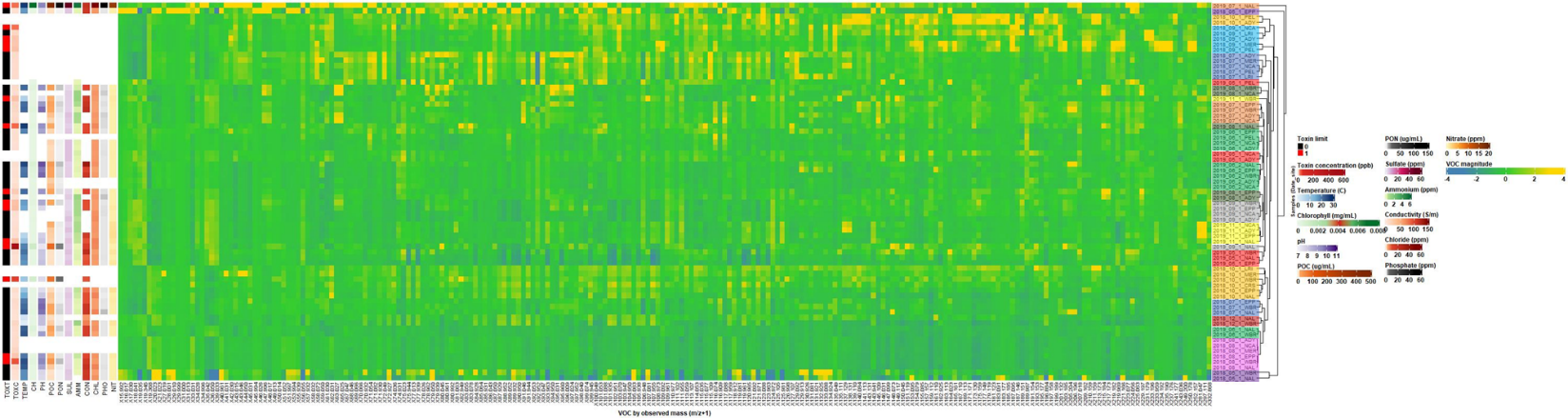


Figure S3. Morphological identification of cyanobacteria collected at UKL. Cyanobacteria identified include *Anabaena/Dolichospermum* (a,b), *Aphanizomenon* (c), *Gloeotrichia* (d), and *Microcystis* (e) are given in the figure. Of particular note are panels (f) and (g), which are examples of samples in which morphology typical of *Aphanizomenon* was observed in abundance, but 16S rRNA analysis of these samples were dominated by *Anabaena/Dolichospermum*. Scale bar is 100 µm.


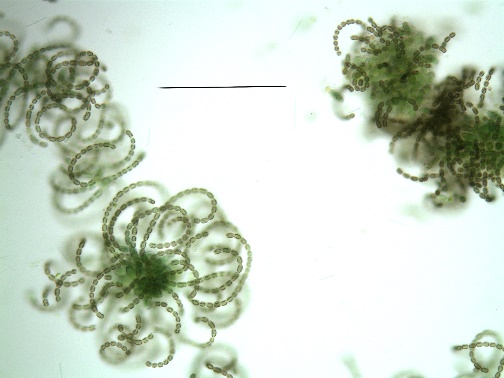

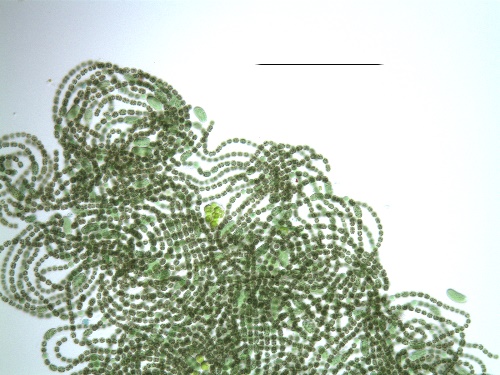

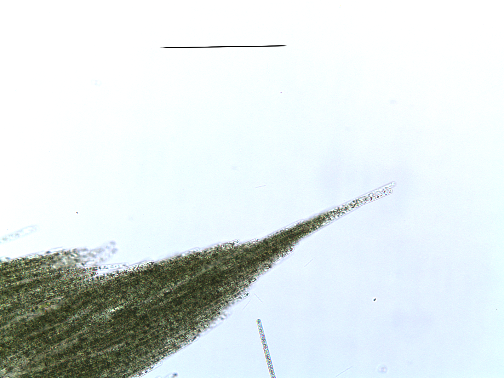


b.

a.

c.


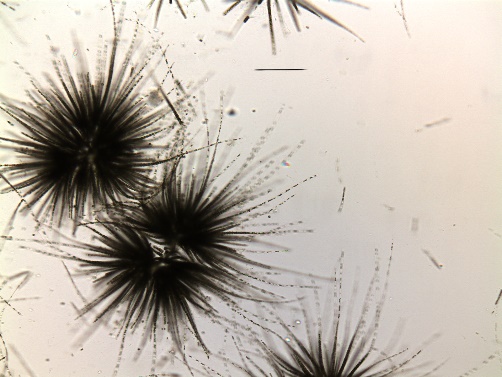

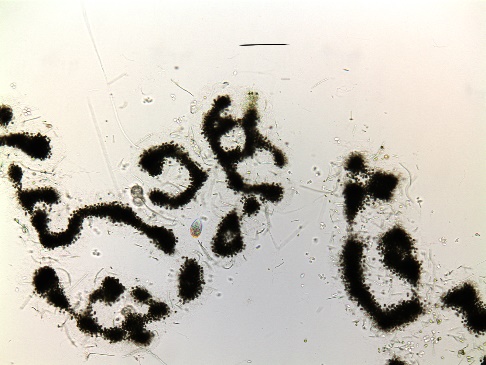

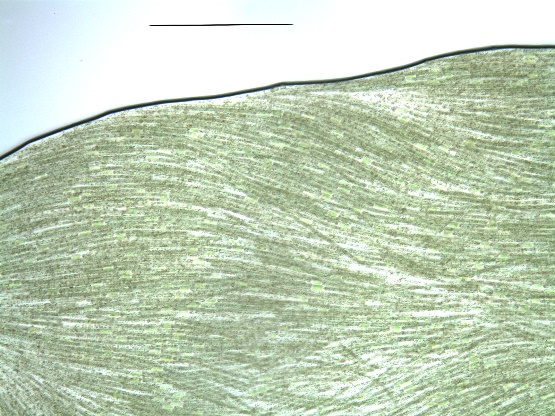


d.

e.

f.


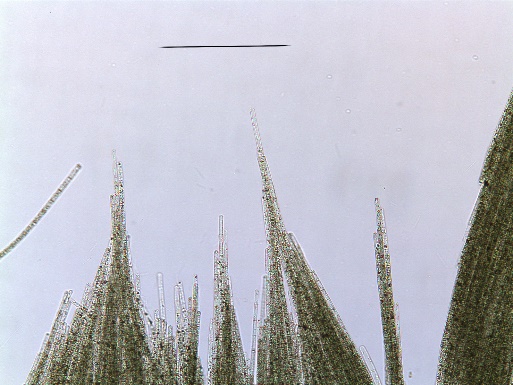


g.

Figure S4. Receiver operating characteristic curves (ROC curves) for the logistical output models: M2 (a), M8 (b), M4 (c), M6 (d). Plots evaluate the diagnostic ability of the logistical regression to predict microcystin concentration ≥0.3 ppb at a probability of 0.5. Area under the curve (AUC) was calculated by averaging the AUCs from the 10 elastic net model runs. Note than in (a), Runs 3 and 6 are on the 50:50 line.


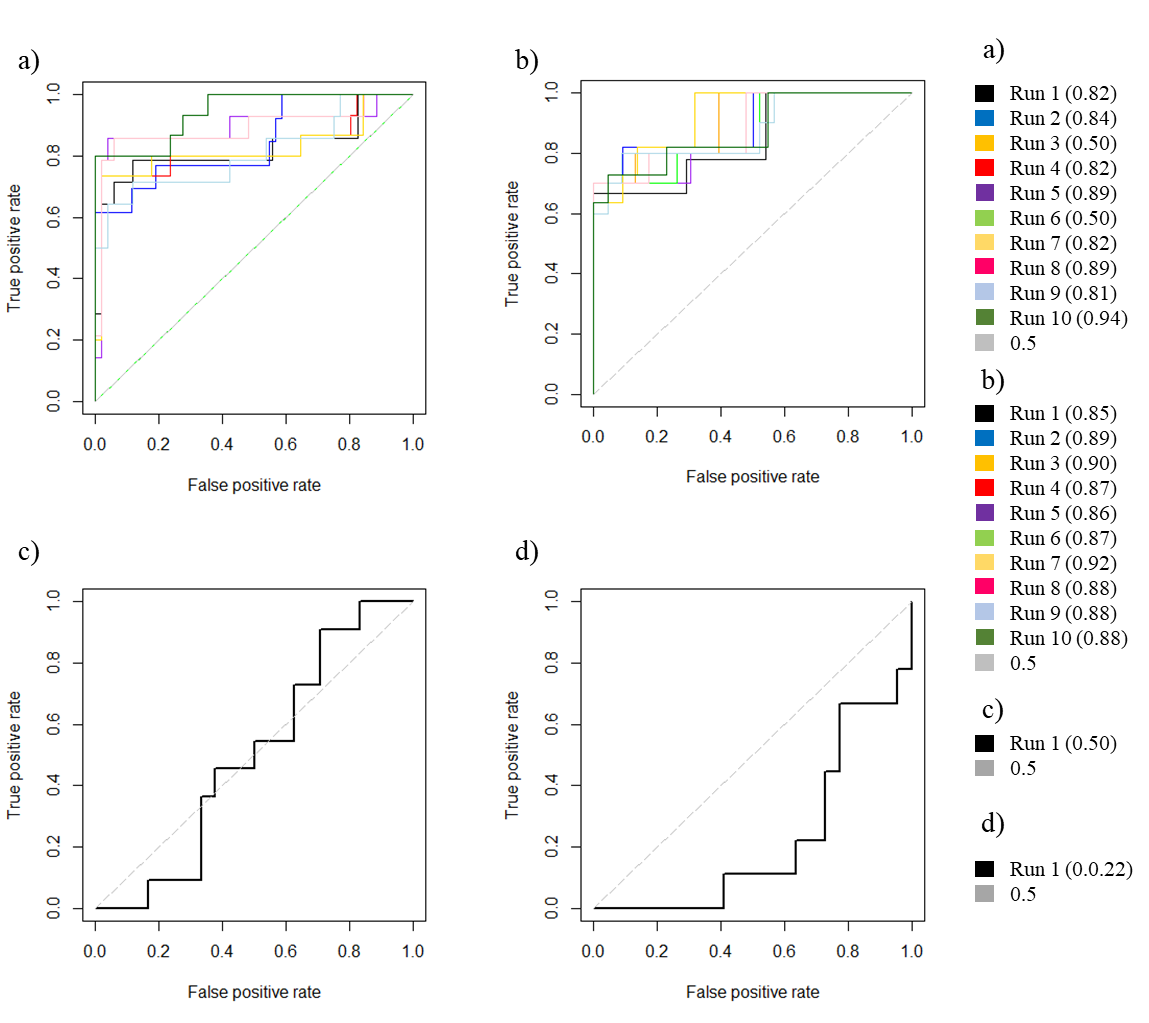


Figure S5. Statistical performances (Mean square prediction error) of elastic net models predicting relative abundances of the four most abundant microbial phyla, classes, and cyanobacterial genera. Error bars are SD.

Figure S6. Pruned tree from Itol used for manually classifying ASVs into their respective groups. Circle location denotes the placement location within the tree, while circle size denotes the number of ASVs placed within that location. Manual classification included extraction of ASVs in each color-coded area within the tree, and reassignment of taxonomy to the genus level. Yellow denotes the ASVs that were identified as *Gloeotrichia*, red are *Anabaena*, green are *Aphanizomenon*, and blue are *Microcystis*. Trees (a) and (b) represent the first and second sequencing runs, respectively.

a.


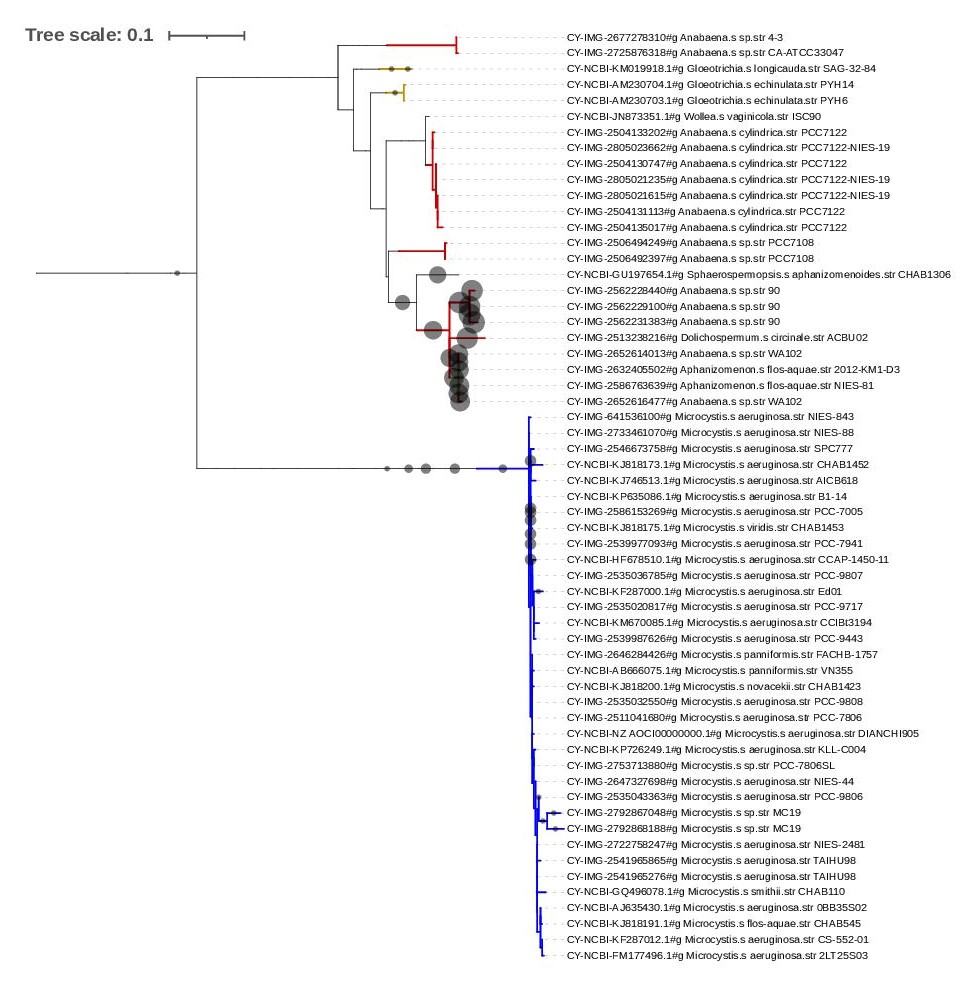


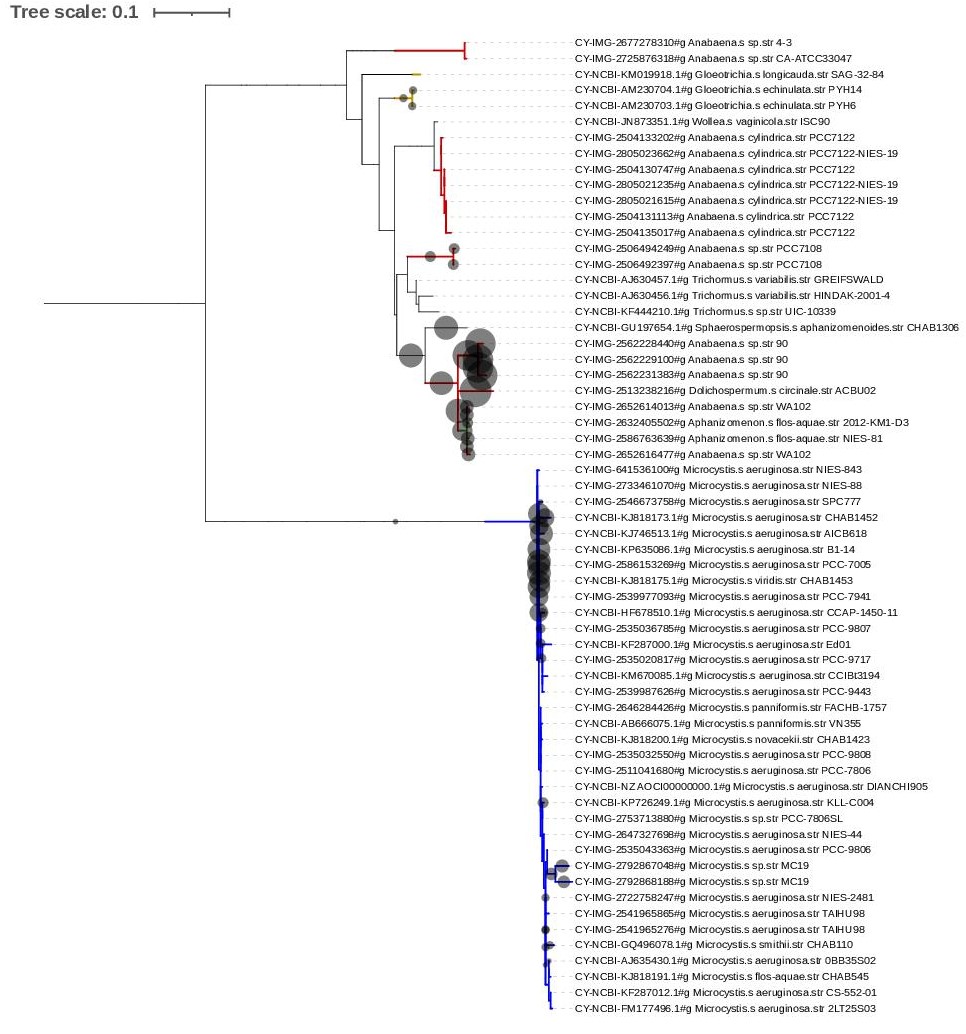


b.
